# Identification of the minimal replicon and the origin of replication of the crenarchaeal plasmid pRN1

**DOI:** 10.1002/mbo3.198

**Published:** 2014-07-25

**Authors:** Silvia Berkner, Mery Pina Hinojosa, David Prangishvili, Georg Lipps

**Affiliations:** 1 Department of Biochemistry, University of BayreuthUniversitätsstr. 30, Bayreuth, 95447, Germany; 2 Molecular Biology of the Gene in Extremophiles Unit, Institute Pasteurrue Dr. Roux 25, 75724, Paris Cedex 15, France; 3 Institute of Chemistry and Bioanalytics, University of Applied Science Northwestern SwitzerlandGründenstr. 40, 4132, Muttenz, Switzerland

**Keywords:** Archaeal plasmid, crenarchaea, minimal replicon, origin of replication, pRN1, *Sulfolobus*

## Abstract

We have determined the minimal replicon of the crenarchaeal plasmid pRN1. It consists of 3097 base pairs amounting to 58% of the genome of pRN1. The minimal replicon comprises replication operon *orf56/orf904* coding for a transcriptional repressor and the replication protein of pRN1. An upstream region of 64 bp that contains the promoter of the replication operon is essential as well as 166 bp of sequence downstream of the *orf904* gene. This region contains a putative transcriptional terminator and a 100 nucleotides long stem–loop structure. Only the latter structure was shown to be required for replication. In addition replication was sustained when the stem–loop was displaced to another part of the pRN1 sequence. By mutational analysis we also find that the integrity of the stem–loop structure is required to maintain the replication of pRN1-derived constructs. As similar stem–loop structures are also present in other members of the pRN family, we suggest that this conserved structural element could be the origin of replication for the pRN plasmids. Further bioinformatic analysis revealed that the domain structure of the replication protein and the presence of a similar stem–loop structure as the putative replication origin are also found in several bacteriophages.

## Introduction

The plasmid pRN1 (5350 bp) has been isolated from *Sulfolobus islandicus* strain REN1H1 (Zillig et al. 1994) and occurs natively together with plasmid pRN2 in its host strain but has been shown to replicate independently of pRN2 (Purschke and Schaefer 2001). It is a member of the pRN family of genetic elements comprising pRN1, pRN2, pDL10, pHEN7, and pSSVx (Keeling et al. 1998; Arnold et al. 1999; Kletzin et al. 1999; Peng et al. 2000). The more recently described plasmids – pTIK4, pTAU4, and pORA1 (Greve et al. 2004) and pIT3 (Prato et al. 2006) – which have been isolated from strains from New Zealand and Italy also contain open reading frames with sequence homology to proteins from the pRN family plasmids. These plasmids, however, are only distantly related to the pRN plasmids which originated from strains in Iceland.

On the whole, our knowledge on the replication of the archaeal plasmids is very limited. Even sophisticated sequence analysis of the plasmidal genomes has only allowed to suggest the replication mode for a few plasmids. In most cases due to the lack of sequence similarity of the putative archaeal replication proteins to well-characterized bacterial or viral replication enzymes neither the replication mode of the archaeal plasmid nor the replication origin can be predicted. Only for some plasmids, for example, pGT5 from *Pyrococcus abyssi* and some small haloarchaeal plasmids, the rolling circle replication could be predicted from the genome sequence and could be verified by experimental evidence. In case of the plasmid pGT5, the double-stranded origin of replication could be determined through the sequence specificity of the initiator protein in an in vitro assay (Marsin and Forterre 1998). In contrast for the remainder of the archaeal plasmids the molecular mechanisms of replication are largely unknown (reviewed in Lipps 2008).

In the case of the plasmid pRN1 biochemical studies on the recombinant plasmid proteins helped to delineate the replication of the plasmid. The plasmid pRN1 has three genes. Two of the genes code of rather small DNA binding proteins. In contrast the third gene occupies roughly half of the plasmidal genome and codes for a 110 kDa protein ORF904. This protein is a multifunctional enzyme with sequence-specific primase activity, DNA polymerase activity, and a weak helicase activity (Lipps et al. 2003). These biochemical activities suggest that ORF904 is directly involved in plasmid replication. The detailed molecular mechanism of plasmid replication and the distribution of tasks between the plasmid encoded and the host proteins is not known. A plausible scenario is that ORF904 recognizes the replication origin and performs the initial unwinding. Then the sequence-specific primase activity of ORF904 could be responsible for synthesizing the initial primers at the opened origin. Next the replication intermediate is handed over to the host replication machinery which could then build up one or two replication forks. Despite of these advances neither the exact mechanism of replication initiation nor the replication origin are known. Typical replication origins of bacterial plasmids are AT rich and contain iterons. On the basis of these characteristics we were, however, unable to identify replication origins neither on the pRN1 plasmid nor on its related plasmids.

In the past it has been speculated that pRN1 is replicated through a rolling circle replication and a double-stranded origin as well as a single-stranded origin have been proposed (Kletzin et al. 1999). Since a sequence-specific endonuclease activity that is required for replication initiation of rolling circle plasmids has not been detected in the plasmidal proteins, it is rather unlikely that the replication of pRN1 proceeds in a rolling circle.

We have suggested that the highly conserved sequence-specific DNA binding protein ORF80 (Lipps et al. 2001) recognizes the replication origin and could then recruit additional factors (such as the plasmidal replication protein ORF904) to the origin. Genetic evidence, however, negated an important role of the ORF80 protein since an interruption of the *orf80* gene is tolerated (Berkner et al. 2007).

In an attempt to better understand the replication initiation of pRN1, we and others (Joshua et al. 2013) used a genetic approach to define the minimal replicon of pRN1. We used a developed shuttle vector system (Berkner et al. 2007) to delineate the minimal replicon of the archaeal model plasmid pRN1. For that purpose we constructed a variety of deletion mutants and tested them for their ability to replicate in the recipient strain *Sulfolobus acidocaldarius* MR31 (Reilly and Grogan 2001). During this work, we identified a noncoding segment of DNA which appeared to be essential for replication. This region could fold into a large stem–loop structure and function as origin of replication.

## Experimental Procedures

### Strains and culture conditions

*Sulfolobus acidocaldarius* MR31 (Reilly and Grogan 2001) was grown in Brock's basal salts medium at pH 3.5 (Brock et al. 1972). Acid-hydrolyzed casein, that is, NZAmine AS (Sigma, St. Louis, USA) (for plates), or enzymatically hydrolyzed casein, that is, tryptone (BD Biosciences, New Jersey, USA) (for liquid medium), were added at 0.1%, d-(+)-sucrose was added at 0.2%. For growth of untransformed cells, 20 *μ*g/mL of uracil was added to the medium. Plates were solidified by addition of 0.6% Gelrite (Sigma) and 10 mmol/L CaCl_2_. Plates and shake flask cultures were incubated at 75°C.

### Transformation of *S. acidocaldarius* MR31

Preparation of competent cells, methylation of plasmids, and electroporation was essentially carried out as described in detail (Berkner et al. 2007). The electroporator used was the Gene Pulser Xcell (Bio-Rad, Hercules, USA) with the following parameters: 1250 mV, 25 *μ*F, 1000 Ω, and 1 mm cuvettes. Regeneration was done for 30 min at 75°C in recovery solution (Kurosawa and Grogan 2005). After regeneration, cells were plated on NZAmine/sucrose plates and incubated up to 11 days at 75°C (see also “assay for replication ability” below).

### Retransformation to check plasmid integrity

Genomic DNA was prepared from *S. acidocaldarius* MR31 and used to transform *Escherichia coli* as described before (Berkner et al. 2007).

### Construction of plasmids

The deletion and point mutation constructs for the delineation of the minimal replicon and the characterization of the origin region were constructed based on the *Sulfolobus–E. coli* shuttle vectors pB, pC, pD, pF, and pL that have been described in detail elsewhere (Berkner et al. 2007). In Figure S1 the vector pC with all relevant restriction sites is shown. The constructs pBdel1, pCdel1 to pCdel3, pDdel1, pFdel1 to pFdel3, pLdel1 to pLdel3, and pCdel26 were constructed by deleting portions of the pRN1 part of the shuttle vectors by already existing restriction sites (Table S1). For the other deletion constructs additional restriction sites were introduced into the shuttle vector by site directed mutagenesis. Primers of 30–45 nt in length were designed carrying the appropriate base exchanges (Table S2). These primers were used in a 10 *μ*L PCR reaction consisting of 1× *Pfu* polymerase buffer, 0.2–0.5 *μ*L of 10 *μ*mol/L forward and reverse primer, 5–50 ng of template plasmid, and 0.3 *μ*L *Pfu* polymerase (Promega, Fitchburg, USA). The PCR program was as follows: 1 min 95°C followed by 16 cycles of 30 sec at 95°C, 1 min at 60°C, and 8 min at 72°C. Subsequently 0.5 *μ*L of *Dpn*I restriction enzyme was added and incubated for 1–2 h at 37°C. The remaining unmethylated plasmids were transformed into RbCl competent *E. coli* XL1Blue cells (Stratagene, La Jolla, USA).

Plasmids were prepared using the Plasmid Miniprep Kit II (Peqlab, Erlangen, Germany) and checked for the presence of the desired mutation by restriction analysis and sequencing. Furthermore, the replication ability of the resulting construct was tested if the constructs were to be further used for the construction of deletion mutants. In case the point mutation(s) were introduced to examine the importance of single bases at least two independent plasmids from two independent rounds of site-directed mutagenesis were tested in *Sulfolobus* to rule out false-negative results due to undetected errors introduced during PCR.

### Cloning of origin regions

**Figure 1:**
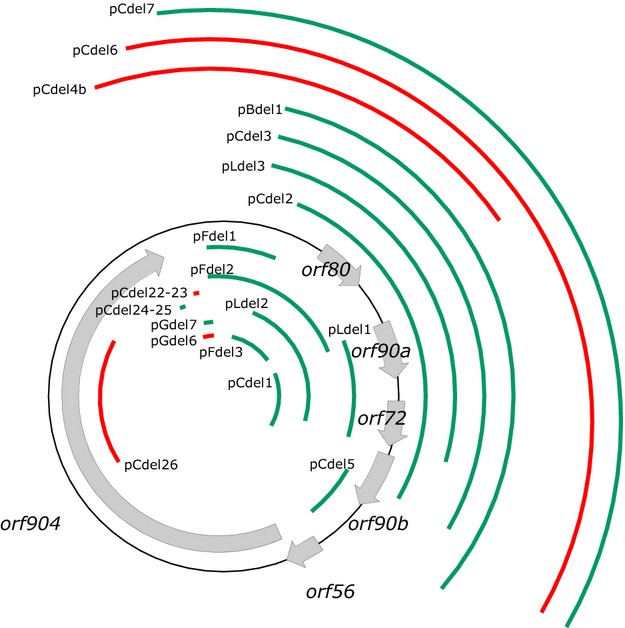
Overview of the deletion constructs of the plasmid pRN1. Based on a series of shuttle vectors (pB, pC, pD, pF, pG, and pL) different parts of pRN1 were deleted while the *Escherichia coli* part of the shuttle vector and the *pyrEF* selection marker were left unchanged. Deletions shown in green did not impair replication, whereas constructs having the parts shown in red deleted were no longer able to replicate in *Sulfolobus acidocaldarius*. The vectors pB, pC, pD, pF, pG, and pL are transposon insertion mutants of pRN1 and contain the cassette comprising the *E. coli* plasmid part and the *pyrEF* selection marker at different positions of pRN1 (Berkner et al. 2007). Examples of shuttle vectors are shown in Figure S1.

The plasmid pCdel6 (Fig. S1), which had been shown to be unable to replicate (Fig.1, Table S1) was used as backbone to construct the pCdel6ori-constructs, by inserting the respective origin regions between the *Sac*II and *Not*I restriction sites of pCdel6.

The primers given in Table S3 with added restriction sites *Sac*II and *Not*I were used in PCR reactions to amplify the different origin regions. The last two numbers of each construct name are derived from the number of the respective forward and reverse primer (Table S4).

### Assay for successful replication

The procedure followed up for all deletion and point mutation constructs to decide on their replication ability is outlined in Figure S2. An example of the plates obtained after electroporation of a viable and a nonviable construct and the retransformation to assess the integrity of the retransformed plasmid is shown in Figure S3.

## Results

### The minimal replicon of pRN1

The minimal replicon is defined as the minimal region of a plasmid sequence that still supports replication of the plasmid. In general, this region may consist of *cis*- and *trans*-acting factors such as proteins and RNA molecules which specifically recognize the origin of replication and are involved in proper regulation of the replication initiation.

For many bacterial plasmids, minimal replicons have been determined by stepwise deletion of plasmid regions and evaluation of the replication ability of the generated deletion constructs. For the determination of the minimal replicon of pRN1, we used a set of shuttle vectors in which the *E. coli* parts were integrated at varying positions into the pRN1 backbone. From these plasmids we deleted increasingly larger stretches of DNA (Fig.1). All constructs (pBdel1 to pLdel3) that were partly or completely deleted for the open reading frames – *orf80*, *orf90a*, *orf72*, and *orf90b* – were able to replicate. Disruption of these four open reading frames by transposon insertion had already shown that these four open reading frames are not essential for the replication of pRN1 (Berkner et al. 2007). By deleting these open reading frames we could confirm our previous observations. Furthermore, the experiments demonstrate that the complete region of the plasmid comprising these open reading frames is dispensable for plasmid replication and that only the replication operon of pRN1 appears to be essential for replication. This operon consists of the two cotranscribed genes – *orf56* and *orf904*. *Orf904* codes for the replication protein of pRN1 (Lipps et al. 2003; Beck and Lipps 2007), whereas ORF56 is involved in plasmid copy number control (Berkner and Lipps 2007). It has previously been shown that an interruption of the replication operon abrogates the replication of pRN1 (Berkner et al. 2007). The eminent question was now, which additional sequence parts of the pRN1 plasmid are essential for successful replication of the plasmid. For that reason several deletion mutant constructs were made to narrow down the region of the minimal replicon step by step (Fig.1).

**Figure 2:**
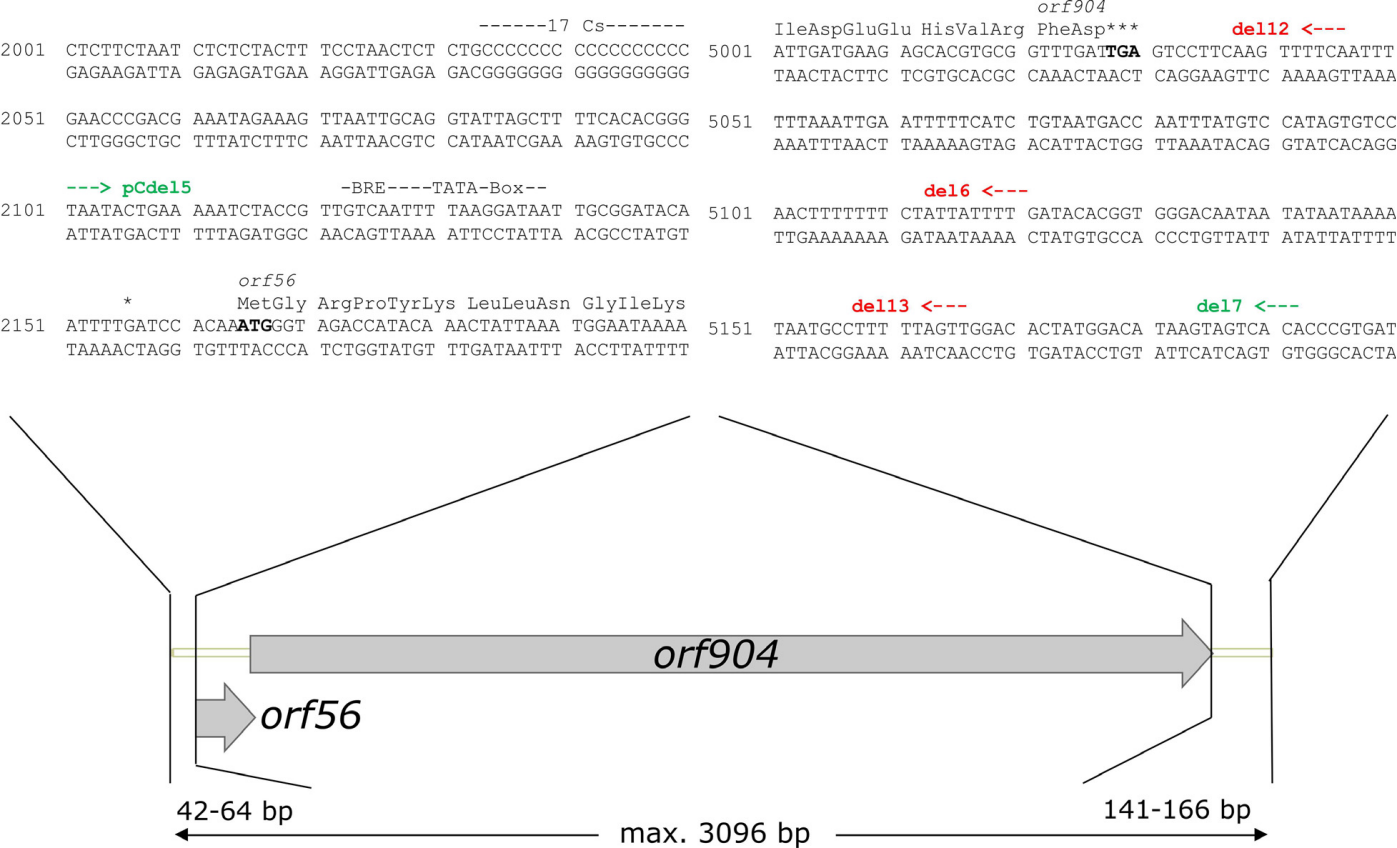
The minimal replicon of pRN1. The start and end of the minimal replicon are shown in detail. Deletion constructs pCdel5 and del7 replicated but del113 did not. End of arrows define the base which was still present in the deletion mutant (red: nonreplicating, green: replicating). Start and end of *orf56* and *orf904* are shown, as well as the BRE and TATA-box, the transcription start site (*) and the C-stretch.

The transcription start site of the cotranscript has been mapped to be 9 nt upstream of the start codon of *orf56* and based on these results a BRE and TATA-box have been identified upstream of *orf56* (Berkner and Lipps 2007). To determine the start of the minimal replicon of pRN1 upstream of *orf56* more precisely a restriction site was introduced directly upstream of the BRE and TATA-box. This construct was not able to replicate. We thus concluded that directly upstream of the BRE and TATA-box no changes in sequence were tolerated. The next sequence change to introduce a restriction site was done 22 bp upstream of the BRE and TATA-box (thus 64 bp upstream of the start codon) and this construct was able to replicate after the mutagenesis procedure. The resulting deletion construct named pCdel5 was obtained after deleting the region more than 64 bp upstream of the start codon was also able to replicate in *S. acidocaldarius* (Figs.1, 2). Remarkably in this deletion mutant a functionally not characterized highly unusual stretch of 17 consecutive cytosines is also deleted. Therefore, absence of the unusual pyrimidine stretch that is also found in other plasmids of the pRN family, for example, in pRN2, seems not to play a role essential for replication of pRN1. Thus, one end of the minimal replicon is located between 42 and 64 base pairs upstream of the start codon of *orf56* (nucleotide positions 2101–2123 pRN1).

To determine the end of the minimal replicon downstream of the replication operon *orf56/orf904* further deletion constructs were made. As the constructs pFdel1 and pFdel2 were still able to replicate the constructs pCdel7, pCdel6, pCdel12, and pCdel13 (Figs.1, 2 and Table S1) which deleted regions even closer to the end of *orf904* were constructed. pCdel7 replicated successfully, whereas pCdel6, pCdel12, and pCdel13 were not able to replicate. The same mutations and deletions were repeated using another shuttle plasmid, pG (Berkner et al. 2007), as starting point. pGdel6 could not replicate, whereas pGdel7 replicated successfully, confirming the observations made with the pC deletion constructs. Therefore, we concluded that the end of the minimal replicon is situated within 25 bp (nucleotide position 5171–5195 in pRN1), thus between 141 bp (pCdel13) and 166 bp (pCdel7 and pGdel7) downstream of the stop codon of *orf904* (Fig.2).

The open reading frames *orf56* and *orf904* had already been shown to be essential for the replication of pRN1 (Berkner et al. 2007). Nevertheless, an *orf904* deletion construct (pCdel26) was tested lacking the part in between the *Mfe*I sites in *orf904* (634 bp of *orf904* deleted) and was found not to replicate.

From these experiments the minimal replicon of the pRN1 plasmid can be narrowed down to 3097 bp. Consequently, only 58% of the pRN1 sequence is absolutely required for plasmid replication. This region consists of the promoter region of the *orf56/orf904* cotranscript, the coding region for the replication protein ORF904, and the copy number control protein ORF56 and a sequence part downstream of *orf904* (Fig.2).

### The region 3′ of the replication protein cotranscript

As stated above, we found that a noncoding region downstream of *orf904* is part of the minimal replicon. We considered two alternatives why the region downstream of *orf904* could be essential for plasmid replication. First, this region could be required for the correct termination of transcription of the *orf56/orf904* cotranscript; second, this region could be part of the replication origin of pRN.

**Figure 3:**
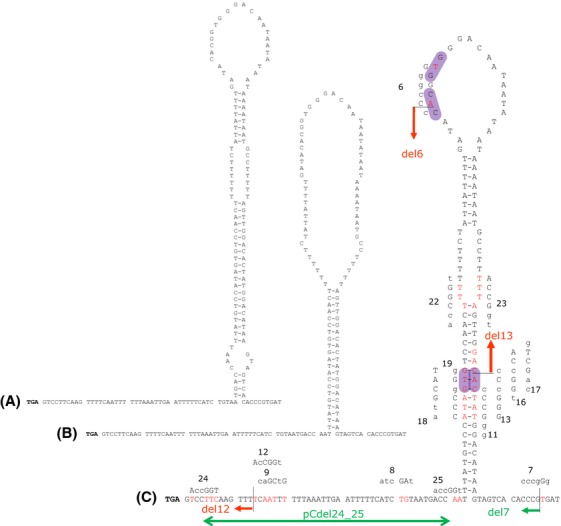
The stem–loop structure. A stem–loop structure was identified within the end of the minimal replicon. The left end of the sequence corresponds to the stop codon of *orf904*. (A) Structure as suggested by Kletzin et al. (1999). (B) Secondary structure predicted by Mfold at 75°C. (C) Secondary structure predicted by Mfold at 37°C. In this structure all point mutations and deletions are depicted. *Deletion mutants*: sequence regions still present in different deletion mutants are indicated by an arrow in red for nonreplicating and in green for replicating constructs. *Point mutants*: overview of the point mutations made within the stem–loop region to test for the function of the putative terminator sequence (construct numbers 9 and 12), for the importance of the GTG/CAC motif (constructs 6, 11, 13, 16, 17, 18, and 19), and to introduce restriction sites to construct deletion mutants (constructs 6, 7, 12, 13, 22, 23, 24, and 25). Exchanged nucleotides are marked in red in the original sequence. The introduced restriction site is shown above/besides the original sequence with exchanged nucleotides marked in capital letters. The GTG/CAC motifs are highlighted in magenta.

It has previously been shown that the cotranscript ends directly downstream of the *orf904* stop codon (Berkner and Lipps 2007). An 8+3 stem–loop structure followed by a T-rich sequence has been identified as putative termination signal. By introducing targeted point mutations into the 8+3 stem–loop sequence we tested whether this putative terminator structure could be the reason for the downstream region of *orf904* to be essential. In the constructs pCqc9 and pCqc12 three, respectively, four nucleotides of the stem part were exchanged to destabilize the putative terminator structure (see Fig.3). Both constructs replicated successfully suggesting that the putative terminator structure is not of relevance concerning the replication of pRN1.

The second possible reason why the region downstream of *orf904* is essential for plasmid replication could be that the origin of replication is situated in this region. We searched for secondary structures and identified a large stem–loop structure starting 54 bp downstream of *orf904* (Fig.3) comprising about 100 bp. The DNA folding program Mfold (Zuker 1989) predicted an additional paired stem at 37°C but at 75°C only a 21 nt stem and a 58 nt loop are predicted. The constructs with the deletion closest to the stop codon of *orf904* that still replicate, pCdel7 and pGdel7, are truncated just downstream of this large stem–loop structure, whereas in the nonreplicating deletion constructs – pCdel13, pCdel6, and pCdel12 – the structure is at least partially affected by the deletions (Figs.1, 3 and Table S1).

**Figure 4:**
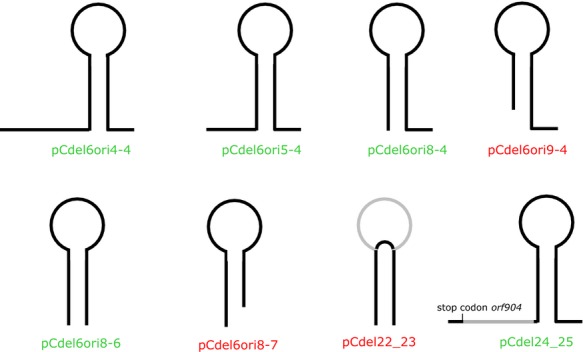
Deletions mutations introducing changes close to the stem–loop structure. The stem–loop is schematically depicted. The respective loop structures were cloned into a nonreplicating deletion construct lacking this stem–loop. Replicating deletion mutants are shown in green, whereas names in red indicate nonreplicating constructs. Two additional deletion constructs, that is, pCdel22_23 and pCdel24_25, were made on the basis of the complete shuttle vector pC. Here, the deleted part is shown in gray. The deletion of the loop is not tolerated, whereas the deletion of the region between the stop codon of *orf904* and the base of the stem including the terminator stem–loop is tolerated.

To test whether this stem–loop structure is a feature that is necessary and sufficient for replication of pRN1, we made use of the nonreplicating deletion construct pCdel6. This deletion mutant contains the whole minimal replicon except the downstream region of *orf904* including the stem–loop structure. Regions comprising the stem–loop structure were PCR amplified and cloned into pCdel6 adjacent to the *E. coli* vector part, relocating the stem–loop structure to a different part of the plasmid (see Experimental Procedures section). By this approach it could be assessed whether the cloned sequence was able to restore the replication ability of the constructs. The constructs pCdel6ori4-4, pCdel6ori5-4, pCdel6ori8-4, and pCdel6ori9-4 were constructed to contain gradually shorter left end flanking sequences (Fig.4 and Table S4). The first three constructs were able to replicate, whereas pCdel6ori9-4 was not able to replicate. This last construct was the only one that did not contain the complete stem–loop structure. The same procedure was followed narrowing down the right-end flanking sequence. The constructs pCdelori8-6 and pCdelori8-7 were tested for that purpose (Fig.4 and Table S4). pCdel6ori8-6 was able to replicate in contrast to pCdel6ori8-7. As for the left flanking region, the first construct showing a deletion in a part of the stem–loop structure failed to replicate.

Thus, all constructs containing the complete stem–loop structure replicated successfully, whereas constructs that were truncated in the stem–loop structure did not produce viable transformants. The construct pCdel6ori8-6 contains only the 100 bp stem–loop without any flanking regions and is able to replicate. Therefore, the isolated stem–loop structure is able to restore the replication ability of a nonreplicating deletion construct.

The importance of the loop region was further assessed with construct pCdel22-23 that contains the stem-forming parts of the structure without the loop region. This construct did not produce viable transformants. With these constructs we could show that the entire 100 bp stem–loop structure comprising the loop is a crucial feature for origin function of pRN1.

With all these mutants the relevance of the region between the stop codon of orf904 and the stem–loop has not been analyzed. We therefore constructed an additional deletion mutant in the wild-type context starting from the complete shuttle vector pC. The region downstream of the stop codon of *orf904* to the beginning of the stem–loop structure was deleted in construct pCdel24-25. This construct was able to replicate successfully confirming our findings obtained with the constructs based on the replaced origin regions and ruling out the possibility that the region in between the end of *orf904* and the start of the stem–loop structure (being still present in nonreplicating background construct pCdel6) is necessary for successful plasmid replication. To sum up, only the 100 bp stem–loop region alone along with the replication operon seems to be necessary and sufficient for successful plasmid replication.

### Sequence analysis of the region downstream of *orf904*

**Figure 5:**
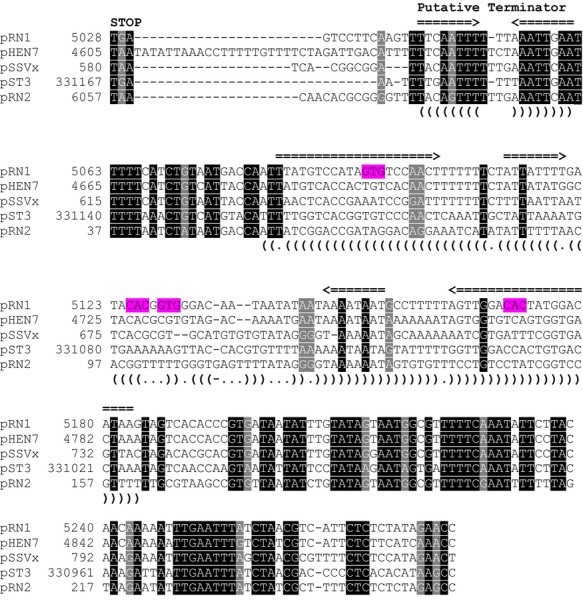
Comparison of the downstream regions of the replication genes. Sequences downstream of the replication gene from the episomal and integrated pRN plasmids were withdrawn from the databases and aligned. Strong sequence conservation was found between all pRN episomal plasmid except pDL10 from *Acidianus ambivalens* and with pST3 integrated into *S. tokodaii*. The sequences were first aligned with T-Coffee. This alignment showed a region of about 100 nucleotides with low sequence conservation flanked by regions of high sequence conservation. The region with low sequence conservation was then analyzed for conserved secondary structure elements with Locarna and structurally realigned. This analysis revealed a highly conserved extended stem–loop structure in all sequences despite its very low sequence conservation. The consensus of this secondary structure as well as the putative stem–loop terminator structure is shown with brackets below the alignment. Arrows above the alignment delineate the stems of the terminator stem–loop and the large stem–loop at 37°C. The sequence alignment starts with the stop codon of the replication protein gene. The sequences GTG and CAC within the pRN1 stem–loop are highlighted.

Having identified the downstream region of *orf904* as essential, we undertook a more detailed bioinformatic analysis of this part of the plasmid. For this purpose we compared the regions downstream of all pRN-type plasmids and included in our analysis also the plasmids pST1 and pST3 integrated into the *Sulfolobus tokodaii* genome (Kawarabayasi et al. 2001) and pXQ1 (Peng et al. 2000) integrated into the *Sulfolobus solfataricus* genome. A multiple sequence alignment of the respective regions showed that in pRN1, pRN2, pSSVx, pHEN7, and pST3 this region is highly conserved. The remaining plasmid sequences, however, could not be aligned very well. Interestingly this region of high sequence similarity is interrupted by a region of very low sequence conservation. Most surprisingly this interior unconserved region corresponds exactly to the stem–loop structure which we have shown to be required for pRN1 replication. We therefore reasoned that a similar stem–loop might also be formed by the other plasmid sequences and undertook a Locarna (structural alignment of RNA sequences) analysis of the central part of the sequence alignment corresponding to the stem–loop structure of pRN1. This analysis clearly demonstrated that although the plasmid sequences are not conserved on the sequence level all the sequences are all able to fold into a highly conserved stem–loop structure (Fig.5).

Our experiments show that the sequence parts downstream of *orf56/orf904* which are highly conserved (i.e., between the stop codon and the stem and downstream of the stem) can be deleted. Possibly these sequence motifs have an auxiliary function and are therefore retained in these plasmids.

It has been shown that the replication protein of pRN1, ORF904, shows primase activity specifically starting at a GTG sequence (Beck and Lipps 2007). We therefore analyzed the stem–loop sequence for occurrences of the sequence GTG and its reverse complement CAC. We find in the stem there is one instance of the GTG/CAC motif and in addition in the loop there is a GTG and CAC motif separated by one nucleotide (Fig.3). Therefore, we tested by mutating the GTG (CAC) sequence motives whether this had an effect on the replication ability. The data in Figure3 show the nucleotides that were exchanged in different mutant constructs. All of the point mutation constructs were able to replicate, when the point mutations were introduced into the wild-type pC shuttle vector. For the construct containing the sequence changes indicated by number 13, changing the GTG complementary CAC sequence in the stem region to CCC, replication was only observed, when the mutations indicated by number 19 restored the perfect symmetry of the stem structure. Other mutations introduced into the stem or loop regions did not interfere with proper replication. Thus we were not able to pinpoint the replication initiation to a single GTG or CAC motif present in the stem–loop structure.

The genetic experiments conducted so far allow us to conclude that the stem–loop structure which is conserved in a number of pRN plasmids is essential for the plasmid and that the stem–loop structure could potentially be the origin of replication. We are, however, not able to present direct evidence that the region 3′ of *orf56/904* functions as origin. Our attempts with two-dimensional gel electrophoretic separation of replication intermediates gave difficult to interpret radiograms with a high amount of supercoiled and intertwined plasmid oligomers even though we used high amount of restriction enzymes and the genomic DNA was well digested as could be judged from the EtBr-stained gels. Possibly the plasmid DNA is especially difficult to digest.

We also investigated whether the replication protein binds sequence specifically to the region downstream of the replication protein gene (Sanchez et al. 2009). However, neither gel-shift experiments nor footprinting experiments (data not shown) indicated specific binding of the full-length replication protein or various deletion mutants to this region. We therefore conclude that additional host proteins might be involved in building up the initiation complex at the replication origin or that complex formation is only possible with negatively supercoiled DNA.

## Discussion

### The minimal replicon and the replication origin

By constructing deletion mutants we could show that the minimal replicon of pRN1 requires the nucleotides 2101–5197 and that in addition the region between the replication protein gene and the stem–loop is dispensable (pCdel24_25, Fig.4). These results are in agreement to the results of a recently published study (Joshua et al. 2013) that showed that the region from position 2101 and 5270 is required. In this study as well as in our study, mutations were generated to change the structure of the stem–loop. Deletions of 18–20 bases of the loop region and deletions of only three bases within the loop yielded plasmids which are no longer able to replicate. Our results are consistent with these experiments as complete deletion of the loop as well as other mutations changing the structure of the stem–loop are not tolerated. Thus, both studies agree that the integrity of the stem–loop is required for successful replication of the plasmid strongly suggesting that the stem–loop harbors the replication origin. In addition, both reports agree that mutations changing the GTG motif in the stem–loop do not abrogate the replication capability of the plasmid.

Nevertheless, both studies come to completely different conclusions concerning how the plasmid is replicated. Joshua et al. (2013) suggest that the stem–loop is the double-stranded origin of rolling circle replication and that the stem–loop directly after the stop codon of the replication protein is the single-stranded origin of rolling circle replication. On the contrary, we suggest that pRN1 is replicated in a different way which appears to be conserved in some bacteriophages (see below) and that the stem–loop directly after the stop codon is a transcriptional terminator. In fact we could show that this part of the plasmid can be deleted.

### Comparison of the origin regions in pRN1 and pRN2

The cryptic plasmids pRN1 and pRN2 have compatible replicons, as both plasmids were originally found together in their native host strain REN1H1 (Zillig et al. 1994). Both plasmids belong to the same plasmid family and have a similar organization of the conserved open reading frames *orf56*, *orf904*, and *orf80* (Lipps 2006). When comparing the nucleotide sequences of both plasmids two separate conserved regions are found: one large region comprising most of the gene of the multifunctional replication protein *orf904* and ∼400 bp of its downstream region and a smaller region around the gene *orf80* and its upstream region. The latter region is not essential for replication as deletion of this region is possible but is required for stable maintenance of the plasmid and might play a role in plasmid segregation to daughter cells upon cell division (Berkner and Lipps 2007). The large conserved region comprises the 100 bp stem–loop structure which was identified as the putative pRN1 origin region. Plasmid pRN2 contains a structurally similar stem–loop of 97 bp (position 61–157 in pRN2, NCBI accession number NC_002101) downstream the open reading frame coding for the replication protein of pRN2. However, the sequence stretch corresponding to the stem–loop in pRN1 and pRN2 exhibits a considerably lower degree of conservation than the flanking regions of the stem–loop (Fig.5). Given the high similarity of their putative origins and their same set of replication proteins it appears that both plasmid replicate using the same replication mechanism. Nevertheless, subtle sequence differences, for example, in DNA binding proteins and in the nucleotide sequences might allow both plasmids to replicate independently of each other.

### Comparison with other origins of replication

Three different modes of replication have been described for plasmids: the rolling circle replicating plasmids, theta replicating plasmids, and a mode of replication with strand displacement. These modes are carried out by different sets of proteins either encoded by the plasmid or by the host genome. In many cases, the plasmid-encoded proteins have an important role in plasmid replication initiation, whereas the further processive replication is carried out by the host proteins. Therefore, DNA polymerases and other components of the replication fork are rarely encoded on a plasmid. In contrast, bacteriophages that are able to produce a large progeny in short time tend to not rely on the host replication proteins but may also encode the respective proteins by themselves. An overview of the different phage replication modules has been reviewed by Weigel and Seitz (2006).

Previously, it had been proposed that pRN1 replicates by a rolling circle mechanism based on the similarity of the above described stem–loop structure to the origin structure of the rolling circle replicating plasmid pLS1 from *Streptococcus agalacticae* (Kletzin et al. 1999). However, this mode of replication is not likely to be used by pRN1, as the characterization of the pRN1 replication protein ORF904 did not reveal any endonuclease activity which would be required for this type of replication initiation.

The activities carried out by the pRN1 replication protein might give a hint how the replication is realized. The pRN1 replication protein has a robust and site-specific primase activity, a DNA polymerase activity devoid of proof reading, and a weak DNA unwinding activity with a translocation direction 3′–5′ on single-stranded DNA and high ATPase activity in presence of double-stranded DNA (Lipps et al. 2003; Sanchez et al. 2009; Beck et al. 2010). As reasoned above, it is more likely that the plasmid-encoded proteins participate in replication initiation than in processive replication. Thus, we suggest that the primase–helicase activity of pRN1 is functionally unrelated to the primase–helicase proteins of bacteriophages, for example, gp4 from T7 (Please refer to Table1 for a comparison of the replication proteins of some model replicons.). In T7 replication the primase–helicase is part of the replication fork and the helicase is encircling the single-stranded DNA lagging strand in 5′–3′ direction helping to unwind the phage genome and the primase is synthesizing with low sequence specificity the primers for Okazaki fragment synthesis (Matson et al. 1983). The same type of activity would be impossible for the pRN1 replication protein as the helicase travels in the opposite direction and is much less processive than the T7 helicase. In fact both helicases are also from different superfamilies: SF3 in case of pRN1 and SF4 in case of T7. Helicases of these two superfamilies assemble as hexameric rings. Especially helicases of superfamily 3 are also able to encircle double-stranded DNA and might also be able to unwind double-stranded DNA at a replication origin, for example, the SV40 protein large T-antigen (James et al. 2004; Hickman and Dyda 2005). In addition, SF3 helicases are structurally similar to ORC proteins which assemble and destabilize the DNA duplex at archaeal and eukaryotic replication origins. Thus, we suggest that the SF3 helicase domain of the pRN1 replication protein is involved in melting the plasmidal replication origin.

**Table 1:**
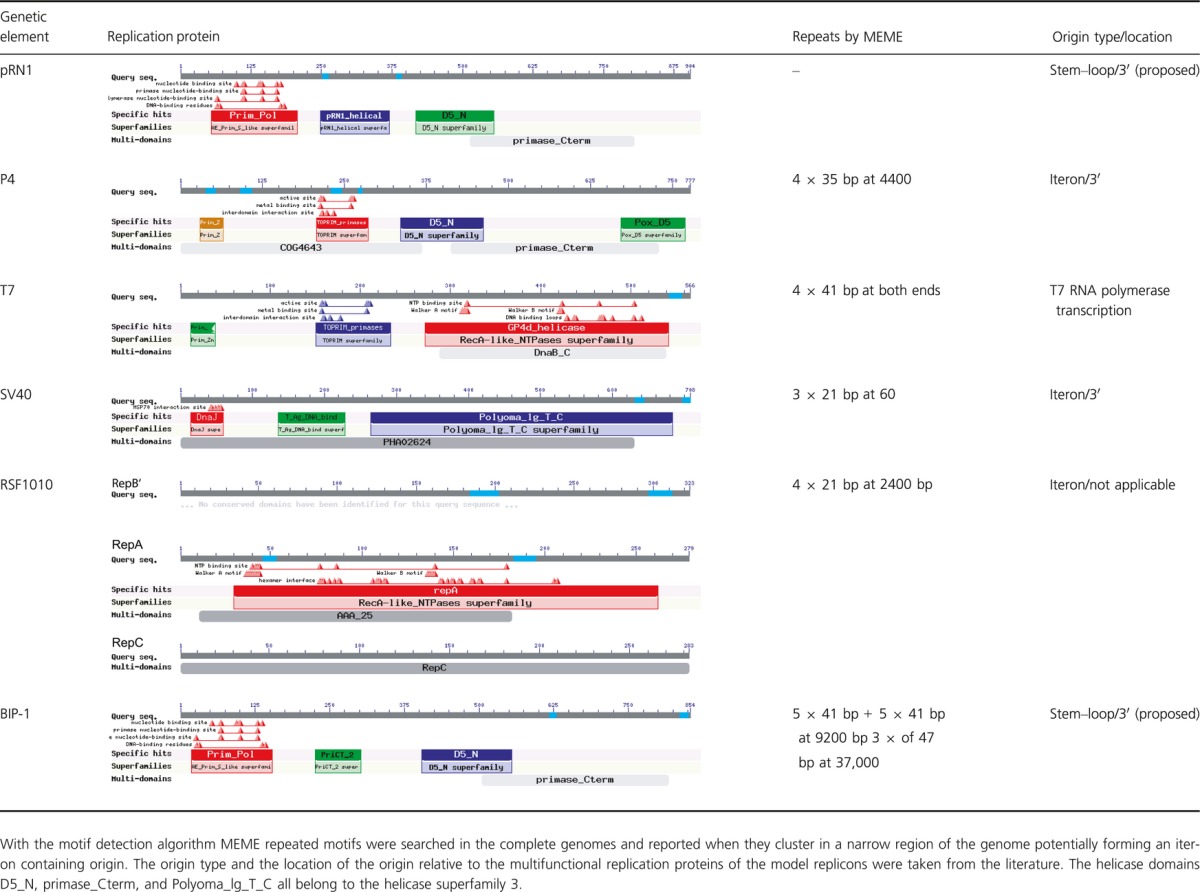
Comparison of the domain structure of plasmidal and viral replication proteins (see text for details).

In addition, the pRN1 replication protein could function similar to the bacteriophage P4 alpha protein. This multifunctional protein binds with a winged helix DNA binding domain at the repeats present at the P4 replication origin followed by unwinding and priming (Ziegelin and Lanka 1995; Yeo et al. 2002). However, two major differences between the P4 and the pRN1 replication remain. The primase of the P4 alpha protein is of the bacterial type (Topprim domain) and the pRN1 replication origin does not have the iteron structure typical for many plasmids, instead pRN1 has a large stem–loop. Remarkably, the pRN1 replication protein also has a winged helix DNA binding domain at its C-terminus as revealed by careful sequence analysis and confirmed by biochemical data (Sanchez et al. 2009).

Another similar replication initiation is found for the RSF1010 plasmid. In this case, however, three separate polypeptides carry out the replication initiation (Honda et al. 1991; Scherzinger et al. 1991; Miao et al. 1995; Geibel et al. 2009). The RepC protein recognizes the repeats of the iteron replication origin. Next, RepA unwinds the double-stranded DNA exposing short stem–loops (*ssiA* and *ssiB*) which are then specifically recognized by RepB' which synthesizes a primer 3′ to the stem–loop. Next host replication proteins extend the primers exclusively in leading-strand mode.

The hallmark of the pRN1 replication protein is its primase activity. We therefore searched the Aclame collection of mobile genetic elements (Leplae et al. 2010) for the presence of primases. We first retrieved from the Conserved Domain database domains with known primase activity and selected one representative protein from each conserved domain. We then queried the Aclame collection using the representative protein, that is, the primase domain of the pRN1 replication protein, the primase domain of the RSF1010 replication protein RepB', and Herpes virus primase as well as the cellular primases of the archeaon *Pyrococcus horikoshii* (small subunit) and *Methanobrevibacter smithii* (large subunit), the bacterium *Synechococcus elongates* and the human Prim/Pol-Protein. Although the Aclame collection contains over 122,000 proteins from 2300 mobile genetic elements, we found only a very limited number of proteins with highly significant (*E* = 0.01) and borderline significant (*E* = 1) similarity to primases. We note, however, that proteins of genetic elements may be subject to rapid divergent evolution. Thus, sequence similarity might be lost between homologous proteins. In total, we only found about 50 proteins half of which are related to the bacterial primase DnaG. Thus, it appears that the involvement of primases in plasmid and bacteriophage replication is indeed minor.

**Figure 6:**
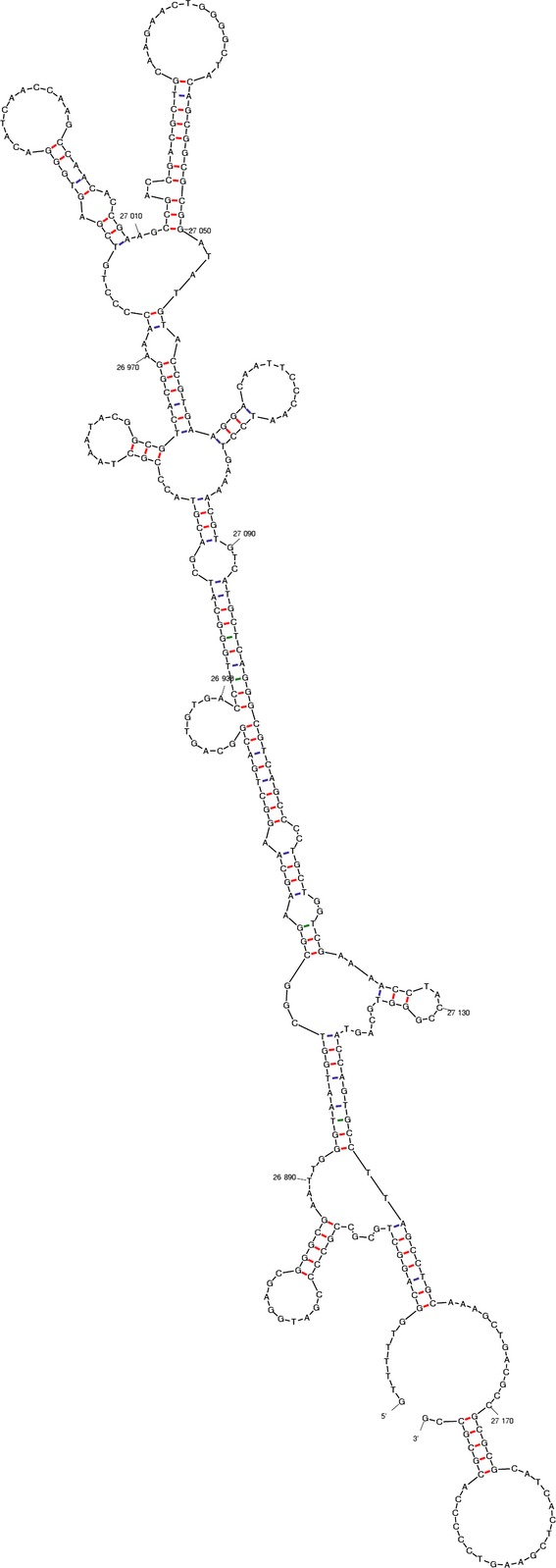
Mfold predicted stem–loop downstream of the BIP-1 replication protein. The replication protein is on the complementary strand and ends at position 27,177 (in the loop of the small stem–loop at the 3′ end).

Remarkably, however, is the similarity of open reading frames from several linear phages to the primase domain of the replication protein pRN1. A more detailed analysis reveals that the replication proteins from the nearly identical phages BIP-1/BPP-1/BMP-1 (*Bordetella bronchiseptica*) are highly related to the pRN1 replication protein. The phage replication proteins have nearly the same domain structure as only the domain pRN1_helical is swapped against the PriCT_2 domain (Table1). The latter domain is also predicted to be helical (Iyer et al. 2005). We analyzed more closely the BIP-1 sequence. With MEME (Bailey et al. 2006), we searched for repeats and found two different repeats with five instances each around nucleotide position 9200 (within a gene coding for a crystalline protein) and another three instances of a 47 bp repeat at position 37,000. Both iteron structures do not appear to be a replication origin. Instead we found 3′ to the replication proteins a large stem–loop as also observed for pRN1 (Fig.6). We therefore suggest that these types of replication proteins carrying an archaeoeukaryal primase domain, SF3 helicase, and a winged-helix DNA binding domain recognize and assemble at a stem–loop structure.

**Figure 7:**
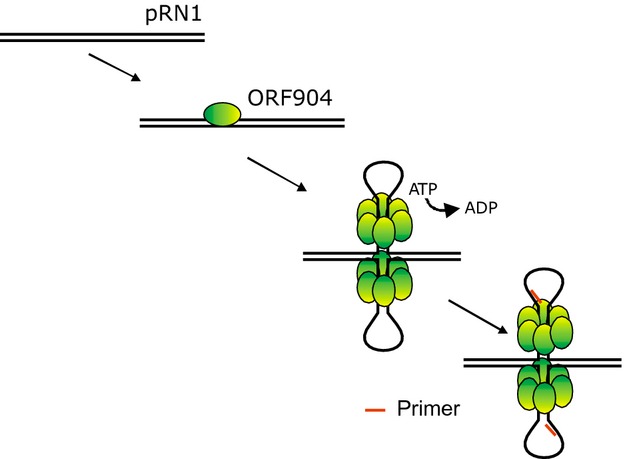
Model of replication initiation involving stem–loops. The replication protein ORF904 binds sequence specifically at not yet identified sequence motifs present downstream of its replication origin. Initial DNA contacts are mediated through the winged-helix DNA binding domain, the beta-hairpins of the helicase domain, and eventually also by the prim/pol domain. Oligomerization occurs and is facilitated by the stem–loop structure and powered by ATP hydrolysis. Primer synthesis may occur at the exposed single-stranded DNA of the stem–loop. Alternatively the helicases “pump” double-stranded DNA widening the loop.

We suggest that the replication initiation proceeds in these following steps. Initial binding of the monomeric replication protein downstream of its own gene. Then, assembly of a multimeric complex aided by DNA which could fold into an alternative structure at the stem–loop. The replication protein is known to assemble into hexameric rings in the presence of double-stranded DNA and the nonhydrolysable ATP analog AMP-PnP (Sanchez et al. 2009). ATP hydrolysis by the superfamily 3 helicase domain of the replication protein could power DNA unwinding and finally the unwound single-stranded DNA is used as template for the primase (Fig.7). It is possible that the helicase once assembled in the two hexameric rings could further translocate and unwind additional DNA stretches. This would explain why the GTG motif within the stem–loop is not required for replication (see above). Further on the host replication proteins come into play. The host proteins might recognize the replication bubble with the primer/templates and build up two replication forks which progresses in both directions. The bidirectional movement suggests itself for symmetry reasons and is more suitable to allow complete replication of the linear BIP-1 phage genome. In phage BIP-1, the putative replication is located at about position 27,000, thus roughly in the middle of the linear genome of 42,638 bp.

During the preparation of the manuscript Joshua et al. (2013) also reported on the delineation of the minimal replicon of pRN1. The group argues that pRN1 is replicated via the rolling circle replication but give no insight how such a replication could be carried out by the replication protein encoded on the plasmid. Rolling circle replication requires an endonuclease which cuts site-specifically within the double-stranded origin (Khan 2000). The replication protein does not have sequence similarity to other rolling circle replication proteins nor to nucleases. Rolling circle replication further requires a single-stranded origin. The *sso* suggested by Joshua et al. is probably a terminator stem–loop and our experiments show that this structure can be deleted. Thus, in our view a rolling circle replication of the pRN1 plasmid is very unlikely.

Here we demonstrate that the stem–loop structure is a conserved feature within the pRN plasmid family and that similar replication modes might also operate in linear phages. Although we cannot present direct biochemical evidence how the replication proceeds we suggest a replication mechanism which is consistent with the known enzymatic properties of the replication protein.

In summary, we find that pRN1 replication is remarkable in several aspects. Only a single multifunctional replication protein appears to be required to initiate plasmidal replication, replication initiation might take place at a large stem–loop structure and surprisingly the same type of replication might be realized by some linear bacteriophages.

## Supporting Information

**Table S1.** Summary of all deletion constructs and point mutant constructs. **Table S2.** Primers used for site-directed mutagenesis. Exchanged nucleotides are shown as capital letters. **Table S3.** Primers used to amplify different origin regions. The atcg was added to improve the restriction of the PCR product. The *Sac*II or *Not*I restriction sites are shown in between vertical lines. **Table S4.** Summary of the origin replacement constructs. **Figure S1.** Map of the shuttle vectors pC and pG as well as the nonreplicating vector pCdel6. The shuttle vectors pC and pG were used as backbones for the construction of point mutants and deletion constructs. Restriction sites that were used for this purpose are indicated. The nonreplicating shuttle vector pCdel6 was used to construct the origin replacement constructs by inserting different origin regions into the *Sac*II and *Not*I sites indicated. *bla*, *β*-lactamase; *pyrEF*, selection marker for *Sulfolobus*, uracil de novo synthesis. **Figure S2.** Schematic overview of the procedure followed to determine the ability of a shuttle construct to replicate. **Figure S3.** Typical results from a replication assay. (A) Example of the results from plating of a replicating point mutant and a nonreplicating deletion mutant. (B) Example for the determination of shuttle vector integrity after retransformation. M, marker; o, original plasmid; r, retransformed plasmid. Digestion was done with *Sac*I.
