# Supplementary material for: Identification of the minimal replicon and the origin of replication of the crenarchaeal plasmid pRN1
Source: Microbiologyopen. 2014 Jul 25;3(5):688–701. doi: 10.1002/mbo3.198 (PMC4234260; doi:10.1002/mbo3.198)
Supplement: Supplementary file 1 [file mbo30003-0688-sd1.docx]

Supplementary Table 1: Summary of all deletion constructs and point mutant constructs.

Supplementary Table 2: Primers used for site directed mutagenesis.

Exchanged nucleotides are shown as capital letters.

Supplementary Table 3: Primers used to amplify different origin regions

The atcg was added to improve the restriction of the PCR product. The *Sac*II or *Not*I restriction sites are shown in between vertical lines.

Supplementary Table 4: Summary of the origin replacement constructs

Supplementary Figure 1: Map of the shuttle vectors pC and pG as well as the non-replicating vector pCdel6

The shuttle vectors pC and pG were used as backbones for the construction of point mutants and deletion constructs. Restriction sites that were used for this purpose are indicated. The non-replicating shuttle vector pCdel6 was used to construct the origin replacement constructs by inserting different origin regions into the *Sac*II and *Not*I sites indicated. *bla*: β-lactamase, *pyrEF*: selection marker for *Sulfolobus,* uracil *de novo* synthesis.

Supplementary Figure 2: Schematic overview of the procedure followed to determine the ability of a shuttle construct to replicate

pCqc23qc22

123

colonies

pCdel22

-

23

0

colonies


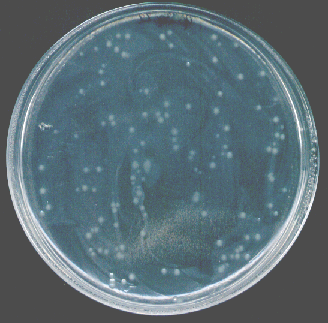

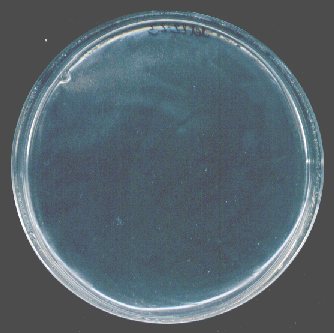

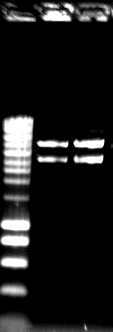


M

o

r

5437 bp

3557 bp

pCqc23qc22

A

B

pCqc23qc22

123

colonies

pCdel22

-

23

0

colonies


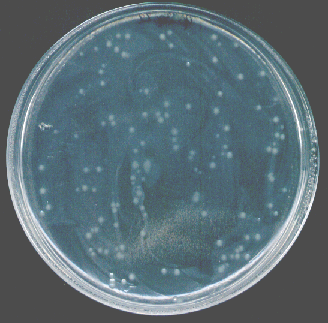

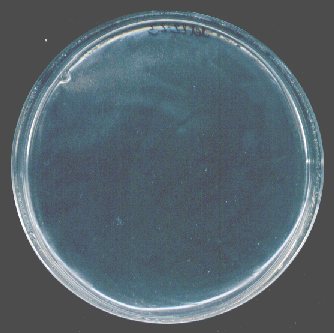

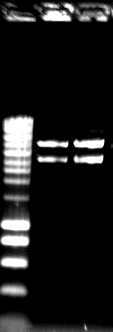


M

o

r

5437 bp

3557 bp

pCqc23qc22

A

B

Supplementary Figure 3: Typical results from a replication assay

A: Example of the results from plating of a replicating point-mutant and a non-replicating deletion mutant. B: Example for the determination of shuttle vector integrity after retransformation. M: marker, o: original plasmid, r: retransformed plasmid. Digestion was done with *Sac*I.
